# Applying Deep Learning-Based Human Motion Recognition System in Sports Competition

**DOI:** 10.3389/fnbot.2022.860981

**Published:** 2022-05-20

**Authors:** Liangliang Zhang

**Affiliations:** Academy of Sports and Leisure, Xi'an Physical Education University, Xi'an, China

**Keywords:** deep learning, human motion recognition, sports, recognition rate, convolutional neural network, data set

## Abstract

The exploration here intends to compensate for the traditional human motion recognition (HMR) systems' poor performance on large-scale datasets and micromotions. To this end, improvement is designed for the HMR in sports competition based on the deep learning (DL) algorithm. First, the background and research status of HMR are introduced. Then, a new HMR algorithm is proposed based on kernel extreme learning machine (KELM) multidimensional feature fusion (MFF). Afterward, a simulation experiment is designed to evaluate the performance of the proposed KELM-MFF-based HMR algorithm. The results showed that the recognition rate of the proposed KELM-MFF-based HMR is higher than other algorithms. The recognition rate at 10 video frame sampling points is ranked from high to low: the proposed KELM-MFF-based HMR, support vector machine (SVM)-MFF-based HMR, convolutional neural network (CNN) + optical flow (CNN-T)-based HMR, improved dense trajectory (IDT)-based HMR, converse3D (C3D)-based HMR, and CNN-based HMR. Meanwhile, the feature recognition rate of the proposed KELM-MFF-based HMR for the color dimension is higher than the time dimension, by up to 24%. Besides, the proposed KELM-MFF-based HMR algorithm's recognition rate is 92.4% under early feature fusion and 92.1% under late feature fusion, higher than 91.8 and 90.5% of the SVM-MFF-based HMR. Finally, the proposed KELM-MFF-based HMR algorithm takes 30 and 15 s for training and testing. Therefore, the algorithm designed here can be used to deal with large-scale datasets and capture and recognize micromotions. The research content provides a reference for applying extreme learning machine algorithms in sports competitions.

## Introduction

With the further penetrating of computer technology (CT) into the sports fields, more CT-empowered approaches are seeing applications in athletes' training, saving the workforce while sharing training experiences. Most commonly, CT can assist coaches and athletes in tactical formulation through video content analysis (VCA) (Jiang et al., 2021). In particular, VCA can often quickly identify the tactical information in the video, thereby improving the efficiency of analytical work. VCA mainly uses image processing technology. Due to the huge amount of information in competitive sports training and the high requirements for the processing ability of machines, the human motion recognition (HMR) method combined with deep learning (DL) is used chiefly in sports VCA (Wang Q. Z. et al., 2021). In terms of human detection, the research is abundant. Many methods have been proposed to quickly and accurately detect people in video images. However, only detecting people is far from enough due to the rising and varying application demands. In many scenarios, it is necessary to further perform motion recognition on the detected people. Therefore, the accurate and real-time HMR in the video image and positioning and motion analysis is vital in real-life scenarios. Specifically, HMR-related technologies are used in traffic scheduling, urban security, gymnastics rehearsal, and stage scene analysis. There are many scenes where target detection, positioning, and motion recognition can greatly improve work efficiency and reduce human resources and material consumption (Li et al., 2021). For example, in group gymnastics rehearsal, it is possible to evaluate the performance of individual members against given standards by detecting and analyzing their positions and movement. Such can improve the overall rehearsal efficiency. The athletes' technical movements are scored using HMR technologies in the Olympic gymnastics' competition. In some interactive games, versatile HMR methods are employed to present a better gaming experience for players. Specifically, virtual reality (VR) games can analyze and recognize the player's movements and intelligently identify the player's instructions. With the introduction of DL's concept, many scholars have devoted themselves to DL research and have made great progress and innovation.

Deep learning is a subcollection of machine learning (ML). It is a new research direction that mimics the human brain to enable machines to cluster data, learn features, and forecast with incredible accuracy. Simply put, it makes computers intelligent. DL is the representation and internal law of ML sample data. Interpreting the information obtained in the learning process help to realize the artificial intelligence (AI) training. The collected information includes images, texts, and sounds. In essence, DL is a kind of ML algorithm (Hsu et al., 2021), which has seen applications in many fields, including personalized technology and data mining (Sahu et al., 2021). Because of the superior processing ability in image understanding, DL algorithms are often used in the field of VCA. As a typical DL model, a convolution neural network (CNN) can realize a multiple-layer DL structure by convoluting and sampling the original image (Khaydarova et al., 2021). Thus, CNN has exerted excellent performance in visual target recognition (Sarma K. V. et al., 2021). In particular, CNN can extract complex patterns with high reference accuracy, suitable for image processing with spatial relationships, such as the DL applications in computer vision (CV) (Jin et al., 2021). CV technology mainly uses computers and cameras to capture, track, and measure the research object. Finally, combined with an AI algorithm, CV realizes automatic motion recognition of the research object. Meanwhile, CV technology solves many shortcomings of traditional human body recognition technology (Liang et al., 2021; Shen et al., 2021). The research of HMR covers multi-disciplinary knowledge, including AI, image processing, and pattern recognition (PR) (Zhang et al., 2021). The HMR algorithm based on multi-feature fusion (MFF) has become mainstream. So far, researchers have designed the HMR algorithm based on the depth-image and obtained a high computational efficiency. But the model performs poorly on micromotion recognition. Then, others have proposed a sequential deep belief network (SDBN)-based online HMR model to extend the deep belief network (DBN) model's recognition ability over static image recognition. However, the SDBN model also prolongs the training time and thus is less time-effective on large-scale datasets. Therefore, HMR design should factor in micromotion recognition performance apart from the time efficiency on large-scale datasets.

**Figure 1:**
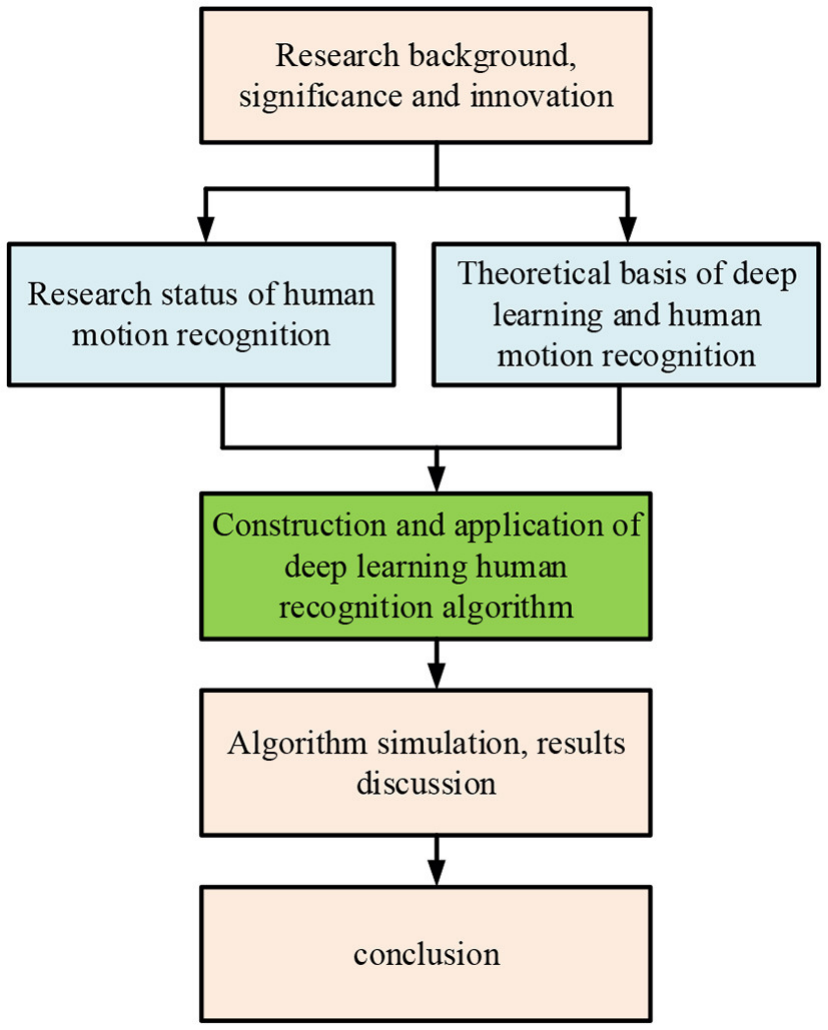
The organizational structure.

The present work will study the application of the HMR system in sports competitions. In particular, HMR in sports competition is tracking and recording human motions through some time-specific key motion points. Then, the key points are expressed by mathematical methods. The application of the HMR system is of great significance to developing competitive sports. Based on the DL algorithm, the present work uses the HMR system to analyze the sport's tactics in sports competitions with high efficiency and quality. Specifically, it introduces the research background of HMR, designs the algorithm considering large-scale datasets and micromotion recognition, and finally evaluates the algorithm's performance through simulation experiments. The innovation of the present work is to apply HMR under DL to the field of sports competition and design a sports video-oriented HMR algorithm using kernel extreme learning machine (KELM) multidimensional feature fusion (MFF) (hereafter, KELM-MFF-based HMR algorithm). The research content provides a reference for developing HMR in sports competition fields. The organizational structure is shown in Figure 1. The Introduction introduces the application background of DL in the field of HMR and proposes the research questions. The literature survey summarizes and analyzes the development of HMR. The HMR algorithm of DL using KELM-MFF is applied to sports competitions. Finally, the algorithm simulation is carried out.

## Literature Survey

The development of human body recognition technology began in the 1990s, and the traditional research direction of HMR is the identification, classification, and characterization of relevant information in sports competitions (Chen and Lee, 2021). Sports information representation includes the description of specific movements of the human body, the detection of spatiotemporal information points in videos, and the tracking and recognition of motion-intense trajectory (IT) (Shieh et al., 2021). There are many ways to characterize specific human motions, such as combining multiple camera videos from different angles. Accurate HMR can be achieved by estimating the motion posture of the human body (Gao et al., 2022). HMR model can be implemented by focusing on specific human parts' motion states. Then, motions can be positioned by the constraint of the tree structure and specific motion information (Hu et al., 2022). Meanwhile, the spatiotemporal and graphical models can be combined to build an integrated HMR framework (Low et al., 2022). Generally, detection tools must be used to detect spatiotemporal information points in the video and describe their features, such as filters and three-dimensional detectors. Common feature descriptors for human motions in VCA include optical flow histogram and directional gradient histogram (Pardos et al., 2022). The first step of tracking and recognizing the motion trajectory is to pre-process and sample the video, then track the motion, and finally get multiple data representation images (Sharif et al., 2022). Afterward, the characterized human motion information needs to be identified and classified. The traditional research of HMR is mainly based on human skeleton information (Miao and Liu, 2021). Studies have shown that motion history point cloud can also describe and recognize actions.

The CV-based method requires simple equipment and is convenient to deploy. It is the main method to study HMR at this stage. It is mainly divided into top–down and bottom–up detection methods. The top–down detection method directly uses the existing detector to estimate the posture of a single person every time. Thus, the detection time is directly proportional to the number of people detected. With the increase of the target human in the image, the detection time of each image also increases, wherein the bottom–up method can separate the target human in the complex image. This method does not directly use the correlation information of other body parts and the global information of others in the image. However, the efficiency is not significantly improved, and the final local correlation needs large amounts of calculation. For example, Chen et al. (2021b) proposed a bottom–up method to associate some detection candidates with a single human body. However, the final detection time was relatively long. Liu (2022) combined the image-pairing score detection method with ResNet, significantly improving the calculation efficiency. However, it took minutes to detect each image, a far cry from real-time detection (Liu, 2022). Detecting the joint points of human posture is a single frame-oriented method. However, motion recognition analyzes the sequential posture set, featuring time-spatial characteristics. Thivel et al. (2022) believed that superimposing and calculating the motion silhouette of the human body could get the motion energy map and motion history map. They matched the two feature maps with the template to realize motion recognition (Thivel et al., 2022). Bu et al. (2022) used scale-invariant feature transform (SIFT) feature to describe motion trajectory. They then used hidden Markov model (HMM) for HMR (Bu et al., 2022). There is also research on the skeleton points-based HMR. These methods are relatively simple with a relatively low recognition rate. Choi et al. (2022) introduced the concept of “entropy.” They proposed an HMR model based on motion energy using a dynamic time warping algorithm to realize HMR (Choi et al., 2022).

As from the past studies, the DL algorithm helps improve the HMR algorithm's efficiency on large-scale datasets. However, the micromotion-oriented HMR algorithm needs more in-depth research to analyze sports tactics better. In particular, the present work uses KELM to combine the manual features of improved dense trajectory (IDT) with the DL features. As such, the proposed KELM-MFF-based HMR algorithm has both advantages of manual features and DL features and can interpret human motion in sports videos from multiple angles.

## Technical Background and Design of the Proposed KELM-MFF-Based HMR Algorithm

### Development of HMR Technology

**Figure 2:**
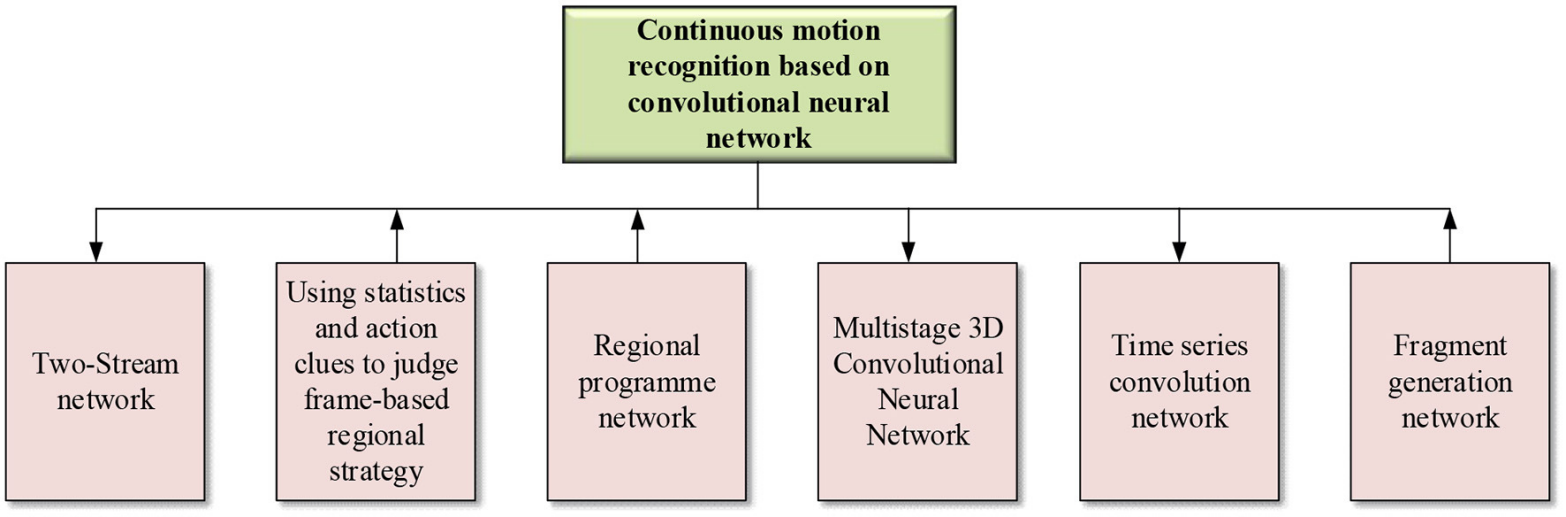
Motion recognition based on CNN.

At present, the most popular HMR system is based on a two-CNN structure (Wang and Feng, 2021), where two CNNs are combined, one for cutting out the action image and the other for inputting the original image. The two-CNN fusion structure reduces the network parameters and accelerate the training speed (Zhang X., 2021). Additionally, some research combines spatial and temporal dimensions of CNNs for HMR. A total of two parallel frames are used to build the CNN. Alternatively, a professional camera is used to accurately recognize the human motion in the video in combination with the long-term recurrent convolutional network (LRCN) (Chen et al., 2021a). Figure 2 displays (Kim S. U. et al., 2021) the content of HMR based on CNN.

Figure 2 implies that with the development of the 3D neural network, 3D CNN sees applications in HMR. Research directions in CNN-based HMR include video frame number, time sequence, region, and other influencing factors.

**Figure 3:**
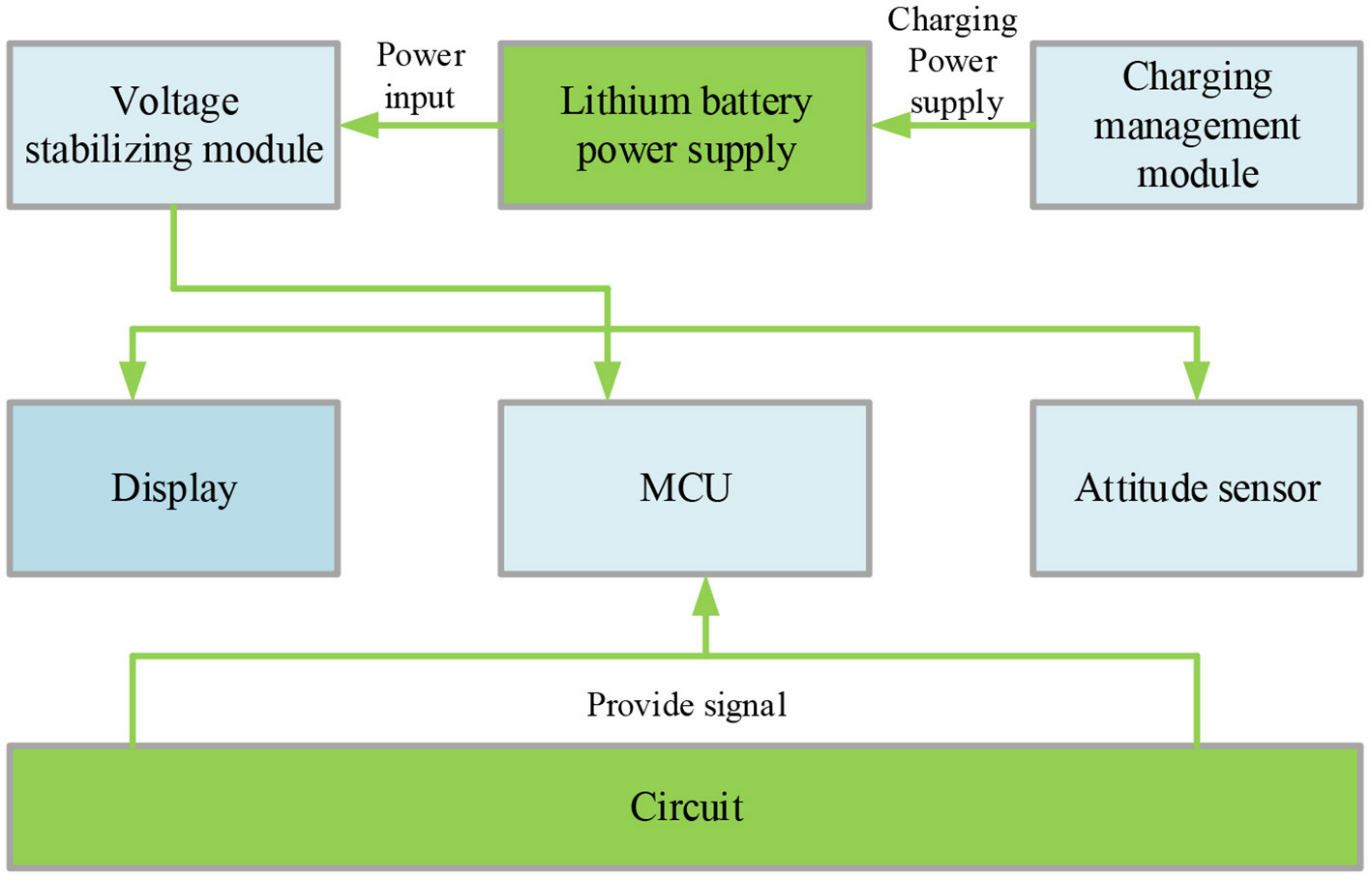
HMR system.

The proposed KELM-MFF-based HMR algorithm comprises a display screen, power supply (PS), controller system, and posture sensor. The system PS is a set of lithium batteries charged by the management module. The voltage stabilizing module provides appropriate PS voltage for the main controller and peripherals. The posture sensor collects the original data and sends them to the microcontroller unit (MCU). Afterward, the MCU sends the processed data results to the screen. The specific process is profiled in Figure 3.

### Design of HMR Algorithm Under DL

The single hidden-layer feedforward neural network (FNN) can be solved by extreme learning machine (ELM) theory, which is more straightforward than other theories. Therefore, the present work selects the ELM to design the HMR algorithm (Hao et al., 2022). Next, the ELM model classifies a certain number of training samples (Su et al., 2021) and outputs as the minimization. The ultimate purpose is to minimize the training error.

Combined with the literature knowledge, Equation (1) gives the compatible expression of KELM.


(1) $$\begin{array}{l} \text{f}\left({\text{x}}_{\text{j}}\right)={\left[\text{P}\left({\text{x}}_{\text{j}},{\text{x}}_{1}\right)\ldots \text{P}\left({\text{x}}_{\text{j}},{\text{x}}_{\text{n}}\right)\right]}^{\text{T}}{\left(\frac{\text{I}}{\text{C}}+\text{P}\right)}^{-1}\text{T} \end{array}$$

In Equation (1), C, *x*_*j*_, and T are the regularization parameter, the training error vector, and the real motion classification. P indicates the kernel function, j = 1, …, n. Equation (1) calculates the significance of the classification attribute of the analyzed motion training video.

**Figure 4:**
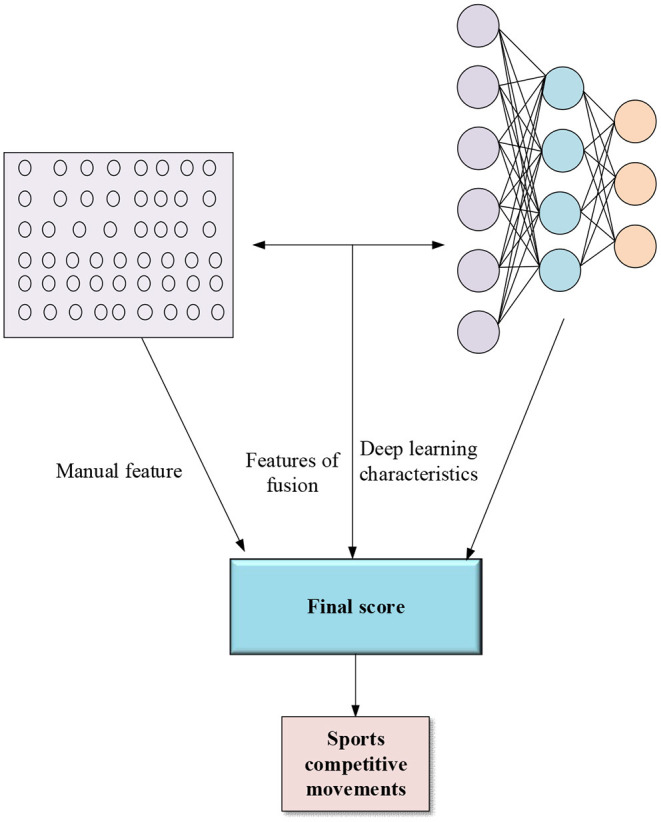
KELM structure.

Dong et al. (2022) found that information fusion helped to improve the algorithm's performance. Based on this, Figure 4 divides the KELM into two parts for analysis.

In Figure 4, KELM first fuses the manual feature kernel and the DL feature kernel and predicts the manual, DL, and fusion feature kernels' score vectors (scores). Then, a neural network-trained classifier classifies the scores.

Equation (2) (Kang et al., 2021) shows the manual and DL features fusion process.


(2) $$\begin{array}{l} \text{P}\left({\text{x}}_{\text{i}},{\text{x}}_{\text{j}}\right)=\text{b}\left({\text{x}}_{\text{i}}\right){\text{b}}^{\text{T}}\left({\text{x}}_{\text{j}}\right) \end{array}$$

Equation (2) expresses the common manual and DL features in the video, where P(x_i_, x_j_) are the different elements of p. The manual and DL feature scores are averaged as the fusion feature scores. Then, scores of fusion, manual, and DL features are estimated.

Subsequently, the three kernel score vectors are used for the fusion operation. The neural network structure calculates the kernel matrix based on the feature scores. Equation (3) is used to estimate the feature scores (Sedmidubsky et al., 2021).


(3) $$\begin{array}{l} \text{P}\left({\text{q}}_{\text{i}},{\text{q}}_{\text{j}}\right)=\text{exp}\left(-\frac{||{\text{q}}_{\text{i}}-\text{q}|{|}^{2}}{{\text{σ}}^{2}}\right) \end{array}$$

Equation (3) is a square exponential kernel expression, where q means the video prediction score, p stands for the Gaussian element, and σ denotes the free parameter.

Further, the proposed KELM-feature fusion-based HMR algorithm is implemented using CNN and manual features. The manual features are coded by the IDT descriptor, including absolute motion features of pixels, description of static features, relative motion features of pixels, and trajectory (Lang et al., 2021). The IDT descriptor uses the Fisher vector and involves a huge amount of data (Kim T. et al., 2021).

Against training data spillover, this section proposes a new mechanism using the principal component analysis (PCA) for the IDT descriptor. The PCA-based new mechanism sets Gaussian element P to 256 to train the model and trains the dataset to 25,600 subsets randomly sampled. Finally, the Fisher vector of the IDT descriptor is obtained. Here, the linear kernel of the descriptor is designed independently, and the descriptive kernel of manual features is solved by Equation (4) (Sarma M. et al., 2021).


(4) $$\begin{array}{l} {\text{P}}_{\text{b}}=\frac{1}{{\text{n}}_{\text{d}}}\sum_{\text{i}=1}^{{\text{n}}_{\text{d}}}{\text{P}}_{\text{i}} \end{array}$$

Equation (4) expresses the kernel matrix of manual features, where n_d_ denotes a descriptor set to 4 types. They are pixel absolute motion features, descriptive static features, relative motion features, and trajectory.

The design of DL features is completed by organizing and processing descriptors. The descriptors of DL features are set to 4,096-dimensional video descriptors, and finally, the kernel matrix PD is obtained by processing (Liu and Ji, 2021).

Equation (5) calculates fusing manual features and DL features.


(5) $$\begin{array}{l} \text{P}=\frac{{\text{P}}_{\text{d}}+{\text{P}}_{\text{b}}}{2} \end{array}$$

Equation (5) is mainly expressed by fusing the average values.

### Characteristics of DL in the Field of Sports Competition

**Figure 5:**
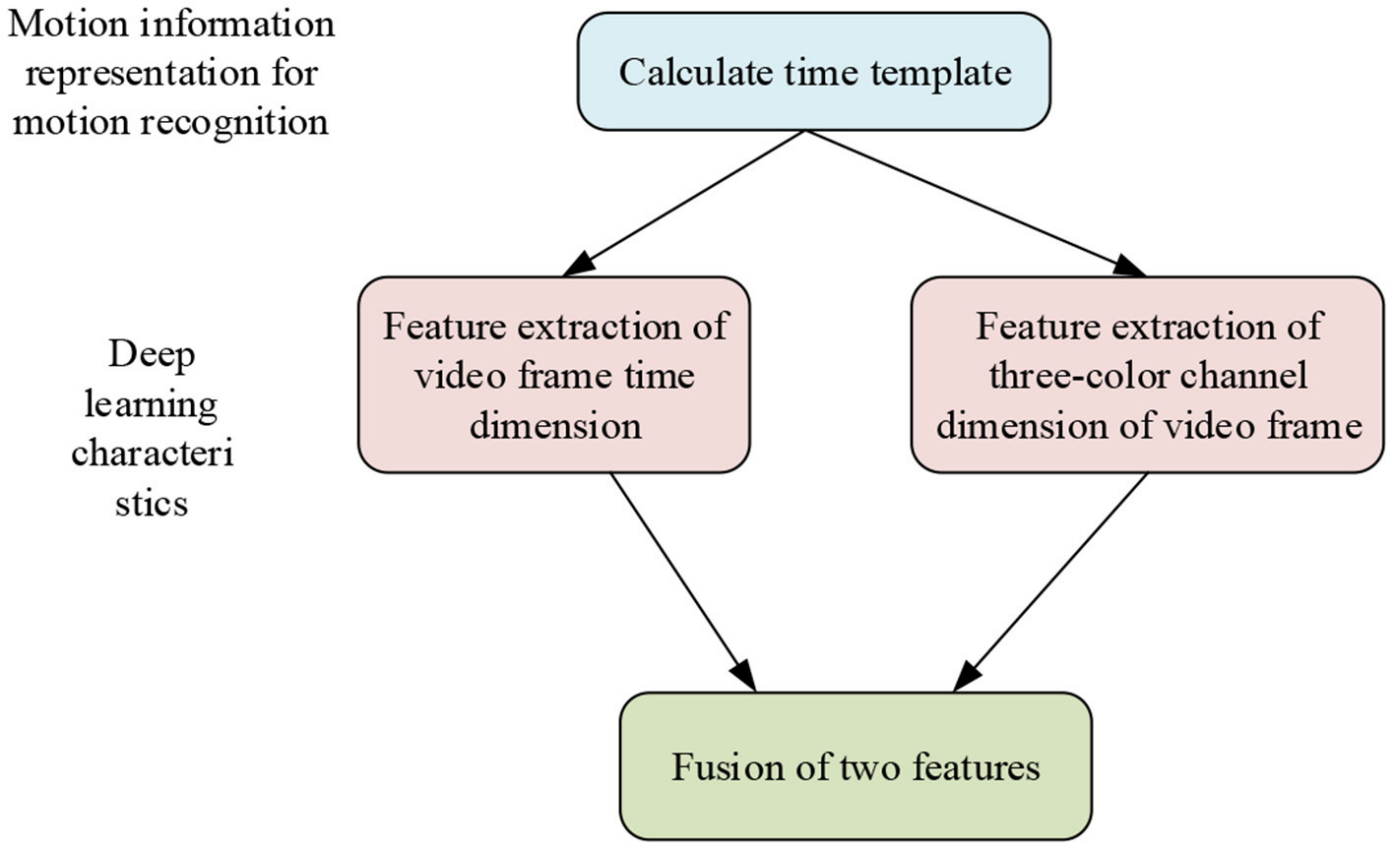
DL in the field of sports competition.

In this section, DL is introduced in the fuzzy judgment of micromotion in sports videos. Figure 5 illustrates the main structure.

Figure 5 indicates that the DL feature extraction is carried out through two dimensions of video: time and red–green–blue (RGB) channels, which will be described later.

Here, the video's time template is used for HMR, the weighted harmonic value of motion information difference, and statistical data at different frames. Equation (6) (Zhang X. Q., 2021) illustrates the specific expression.


(6) $$\begin{array}{l} \text{TT}=\left(\frac{1}{250}\right)\sum_{\text{i}=2}^{\text{n}}{\text{v}}_{1}\cdot \text{r}\left(\text{i}\right) \end{array}$$

The weighted harmonic value in Equation (6) varies between 0 and 250. v means the weight value, n stands for the number of video frames, and r denotes the motion information of each frame.

Equation (7) is obtained by transforming Equation (6).


(7) $$\begin{array}{l} \text{TT}=\sum_{\text{i}=2}^{\text{n}}\left(\frac{{\text{v}}_{1}}{255}\right)\cdot \text{r}\left(\text{i}\right) \end{array}$$

Equation (7) is transformed with fuzzy membership function (MF) to generate Equation (8) (Wang et al., 2021b).


(8) $$\begin{array}{l} \text{TT}=\sum_{\text{i}=2}^{\text{n}}\text{λ}\left(\text{i}\right)\cdot \text{r}\left(\text{i}\right) \end{array}$$

In Equation (8), λ denotes a fuzzy MF, and λ ϵ (0-1). Equation (8) expresses that the weight and fuzzy MF directly affect the significance of sports information. Equations (9–12) display the fuzzy MFs designed here.


(9) $$\begin{array}{lll} \text{λ}1\left(\text{i}\right) & = & 1 \end{array}$$

(10) $$\begin{array}{lll} \text{λ}2\left(\text{i}\right) & = & \frac{\text{i}}{\text{n}} \end{array}$$

(11) $$\begin{array}{lll} \text{λ}3\left(\text{i}\right) & = & 1-\frac{\text{i}}{\text{n}} \end{array}$$

(12) $$\lambda 4\left(\text{i}\right)=\{\begin{array}{c} \frac{2i}{n},\;0i\leq \frac{n}{2}\\ 2-\frac{2i}{n},\frac{n}{2}i\leq n \end{array}$$

In Equations (9–11), *i* ϵ (0, n).

Notably, the membership degree (MD) of the four fuzzy MFs is given in the Section Results.

The CNN is used to describe the DL features of motion information. As mentioned above, CNN is used to learn information features based on time templates. Table 1 lists the parameters of the DL algorithm set.

**Table 1:** DL parameter settings.

**DL**	**Specific parameters**
CNN	2 × 2 feature kernels in the pooling layer and 5 × 5 feature kernels in the convolution layer
Tri-color channel	RGB mode

Table 1 signifies that the architecture of the CNN adopted is 5C-2s-5c-2s, where 2s indicates that the number of feature kernels under the maximum pooling layer is 2 × 2. 5c indicates the number of feature kernels under the convolution layer, which is 5 × 5. Tri-color channel mode refers to RGB mode, applied to SVM to recognize competitive sports motions (Chen K. Y. et al., 2021).

### Simulation Experiment of the Proposed KELM-MFF-Based HMR Algorithm

Subsequently, this section evaluates the proposed KELM-MFF-based HMR algorithm. The experimental sample adopts two kinds of video datasets. The first dataset contains large amounts of low-resolution data, which is used to test the proposed algorithm's large-scale data processing ability. The second dataset has high-resolution micromotion samples. It tests the proposed algorithm's micromotion recognition ability. In this way, sports micromotions in competitive sports can be accurately identified.

#### Experimental Dataset

The datasets used include the (University of Central Florida (UCF) 101 and NATOPS datasets. UCF 101 dataset is collected on the Internet, with high complexity and obvious background clutter. UCF 101 contains 13,320 video clips with 101 action categories. At the same time, this article determines three training set-test set partition schemes. The test dataset selects seven video sequences from 25 groups for each partition scheme. The other 18 video sequences are selected for training.

NATOPS video dataset contains high-resolution images. The motion recognition accuracy of the algorithm designed in this article is evaluated by small hand movements in 24 sports fields. Some gesture movements also include handshape changes. The dataset can be used to evaluate the recognition rate of the action recognition algorithm. The video dataset has a high resolution of 320 × 240; overall, 20 categories are designed. Each category includes 24 small hand movements and 24 × 20 actions altogether. Then, the first five categories are selected to test the algorithm. The last 10 categories are used to train the algorithm.

In the first dataset, the recognition performance of the proposed algorithm is evaluated by comparing it with other algorithms. The algorithms involved include an action recognition algorithm in the context of Converse3D (C3D), an action recognition algorithm in the context of the combination of motion information and SVM (SVM-MFF), an action recognition algorithm in the context of CNN, an action recognition algorithm in the context of IDT, and action recognition algorithm in the context of CNN + optical flow (CNN-T). The algorithm recognition performance evaluation under the second dataset selects 64 × 48 frames as the time template. It extracts the features of the four fuzzy FM functions in Equations (9–12) and then compares them with other algorithms. A total of 6 microhand motion recognition algorithms are involved in this experiment.

#### Recognition Rate of the Proposed KELM-MFF-Based HMR Algorithm

**Figure 6:**
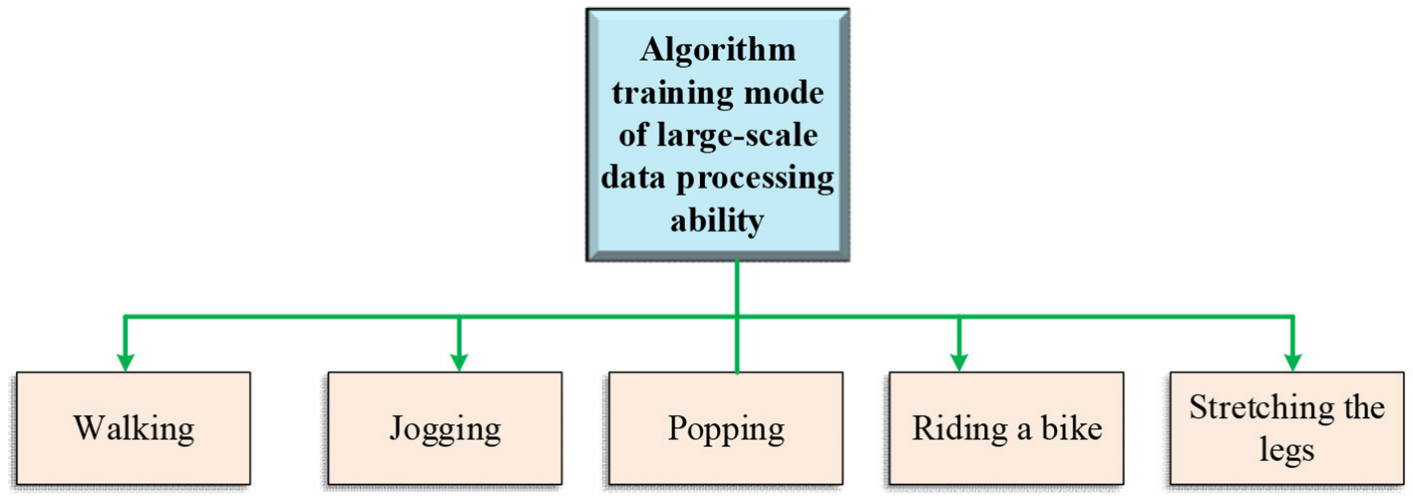
Training mode of large-scale data processing ability algorithm.

The general training framework of the proposed KELM-MFF-based HMR algorithm on the large-scale dataset is outlined in Figure 6.

As signified in Figure 6, the large-scale data processing ability of the proposed KELM-MFF-based HMR algorithm is mainly studied through the motions of jogging, walking, cycling, and stretching legs.

**Figure 7:**
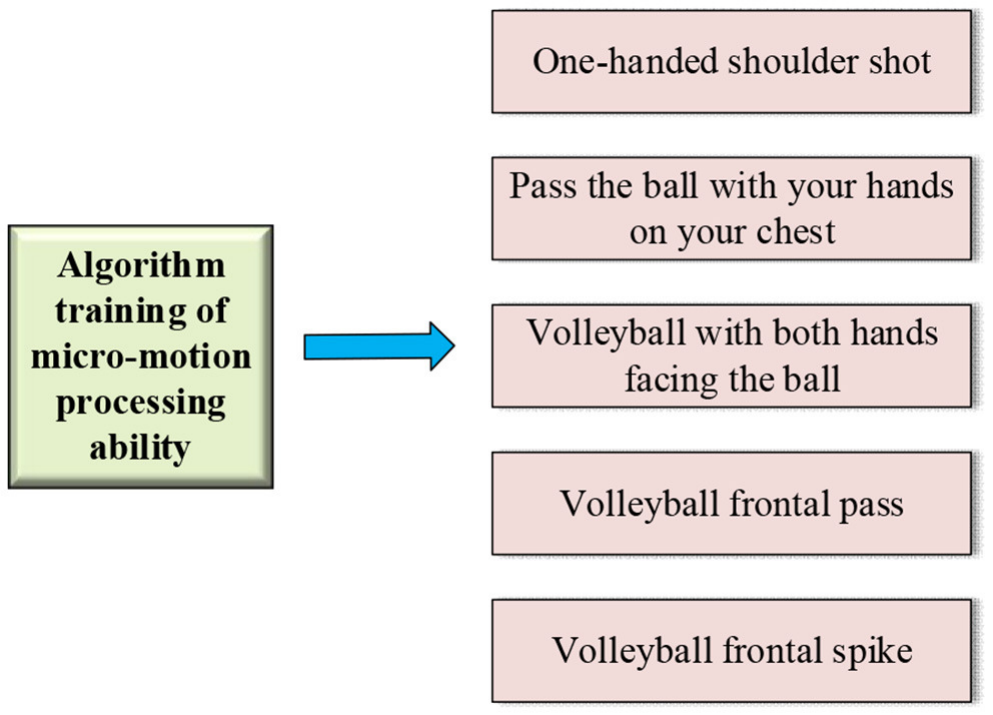
The proposed KELM-MFF-based HMR algorithm's training mode of micromotion processing ability.

The general training framework of the proposed KELM-MFF-based HMR algorithm on the second dataset is portrayed in Figure 7.

As in Figure 7, the second dataset aims to train the micromotion processing ability of the proposed KELM-MFF-based HMR algorithm and focus on hand motions. Therefore, the experiment selects the videos of professional basketball and volleyball games, which both have specific requirements for players' ball-handling skills and thus involve many microhand motions.

#### Algorithm Feature Fusion Strategy

**Figure 8:**
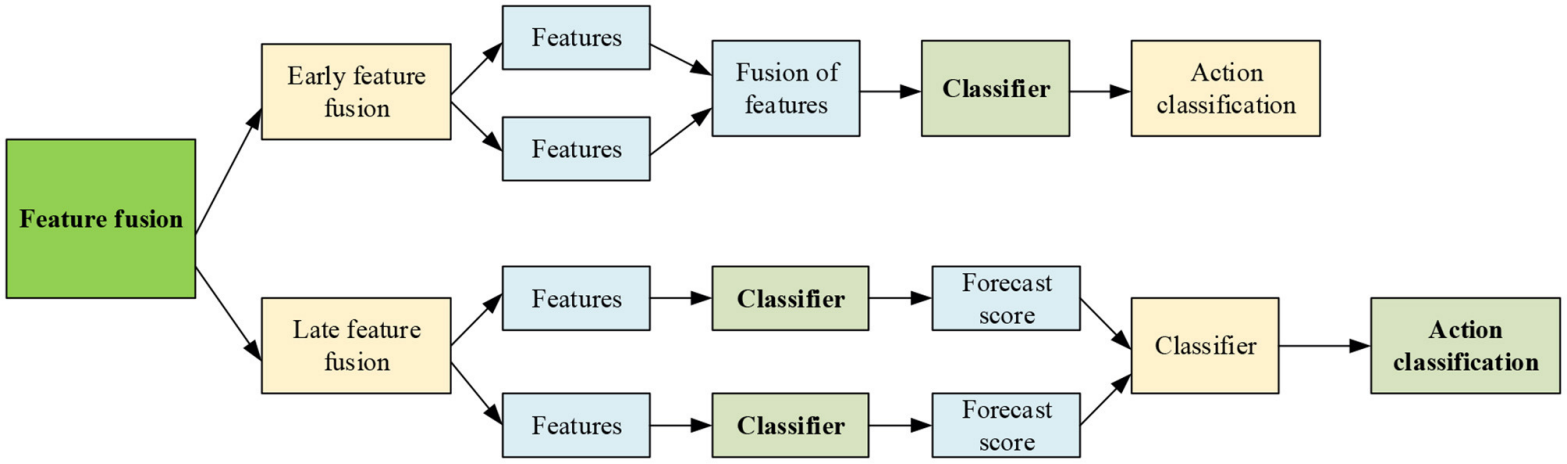
Block diagram of feature fusion.

The feature fusion strategy is divided into two parts: early feature fusion and late feature fusion. The first dataset is mainly used for experimental analysis. Figure 8 displays the main flow of feature fusion.

From Figure 8, the early fusion means feature fusion before classifier, and the later fusion is the feature fusion after classifier. More precisely, the early fusion strategy fuses the feature kernel before the classifier classifies the features. By comparison, the late fusion strategy first fuses the score vectors of each feature. It then classifies the score vector to achieve motion classification. Then, the influence of different feature fusion strategies on the performance of the HMR algorithm is evaluated on the UCF101 dataset. The proposed KELM-MFF-based HMR algorithm is compared with other kernel-based MFF HRM algorithms. Notably, the experiment mainly compares the performance of the proposed KELM-MFF-based HMR algorithm with the SVM-MFF-based HMR algorithm in terms of recognition rate under different feature fusion strategies.

Finally, the time efficiency of the proposed KELM-MFF-based HMR algorithm is evaluated on the UCF 101 dataset with the SVM-MFF-based HMR algorithm. The experimental environment is configured with an Intel i7 3.3 GHz CPU and 16GB RAM.

## Analysis of Simulation Results

### Comparison of Results of Recognition Rate of Different Algorithms

**Figure 9:**
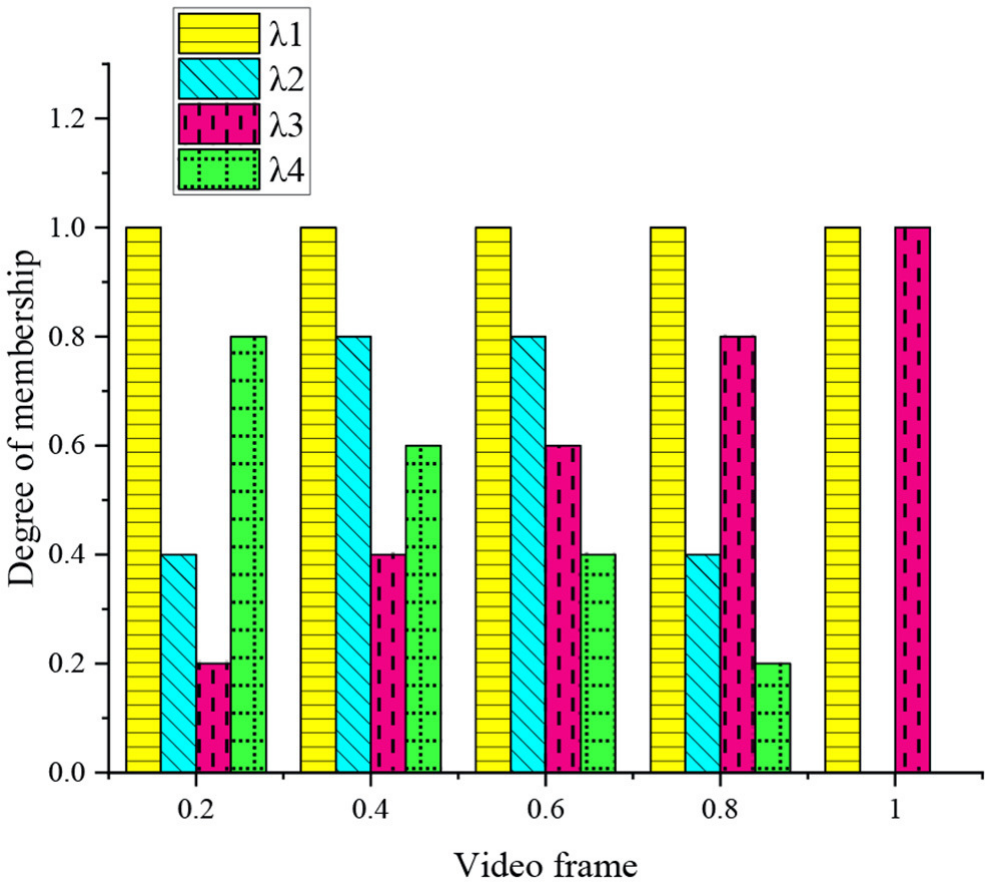
MD of fuzzy MF.

The MD of the fuzzy MF in the previous section is counted in Figure 9.

Figure 9 implies that λ2, λ3, and λ4 represent the beginning, middle, and end regions of sports competitive video in the time dimension. λ1 will not change with the change of video frame. λ1 is mainly used to calculate the energy in sports competitions, which is not affected by time and space. λ2 increases linearly with the change of video frame, which is mainly used to calculate the historical image of sports competitions and is significantly related to the number of video frames. λ3 and λ2 change in opposite directions, and λ3 is significantly correlated with the number of video frames. λ4 assigns the highest significance in the middle area.

**Figure 10:**
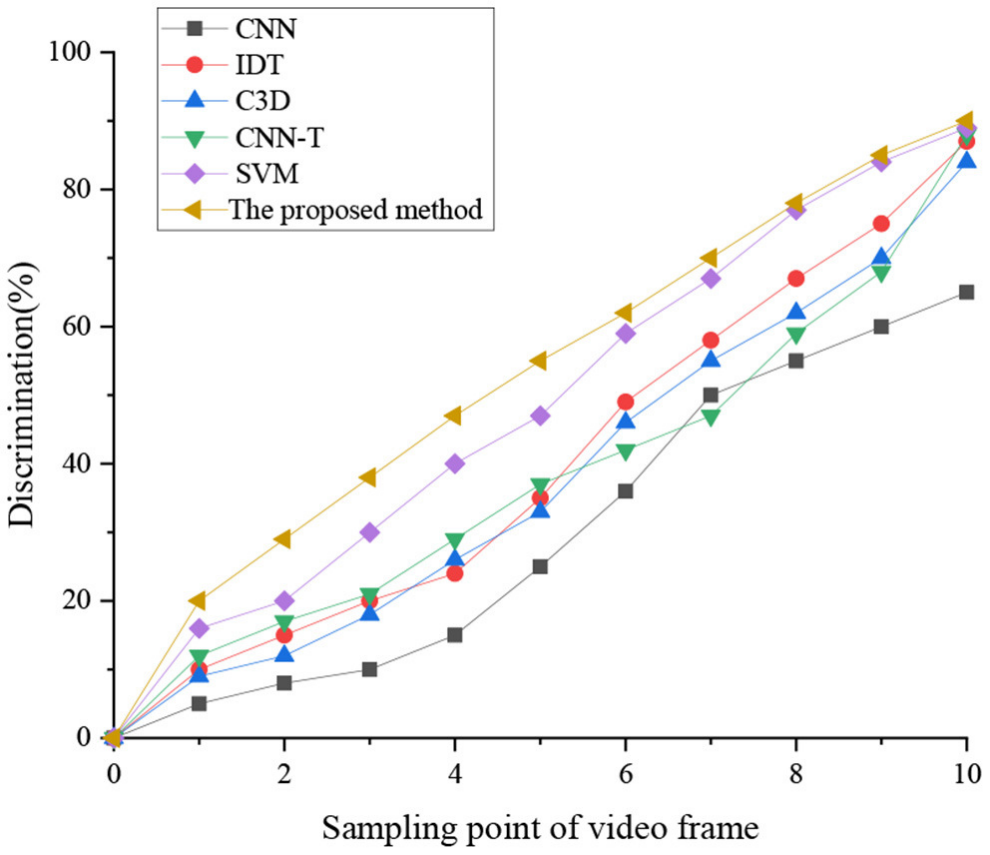
Comparison of large-scale data processing capabilities of different algorithms.

Next, the performance of several HMR algorithms on large-scale data is comparatively analyzed, including the C3D-based HMR, SVM-MFF-based HMR, CNN-based HMR, IDT-based HMR, and CNN + optical flow (CNN-T) HMR. The results are signaled in Figure 10. The present work has not considered the hyperparametric adjustment and only sets the learning rate to 0.01 for all algorithms.

Figure 10 implies that different algorithms have different recognition rates on different superimposed motions. The recognition rate of the proposed KELM-MFF-based HMR algorithm is higher than that of the other five algorithms. The recognition rates of other algorithms at 10 sampling points are sorted from high to low as SVM-MFF-based HMR, CNN-T, IDT, C3D, and CNN. Probably, it is because SVM-MFF-based HMR and CNN-T are MFF algorithms. By comparison, IDT, C3D, and CNN are single feature recognition. The conclusion can be drawn that the recognition ability of the MFF algorithm is better than that of a single feature recognition algorithm. The recognition ability of SVM-MFF-based HMR is not much different from the proposed KELM-MFF-based HMR algorithm. Presumably, the reason is that the SVM-MFF-based HMR algorithm adds a sports information mechanism using a time template to the SVM algorithm, improving the recognition rate. So far, numerous pieces of the literature have shown the MFF algorithm's advantages. Additionally, the present work results are consistent with Tanaka et al. (2022) latest research results. The recognition ability of MFF is stronger than that of a single feature recognition algorithm. The difference is that many comparison models are used in the present work (Tanaka et al., 2022).

**Figure 11:**
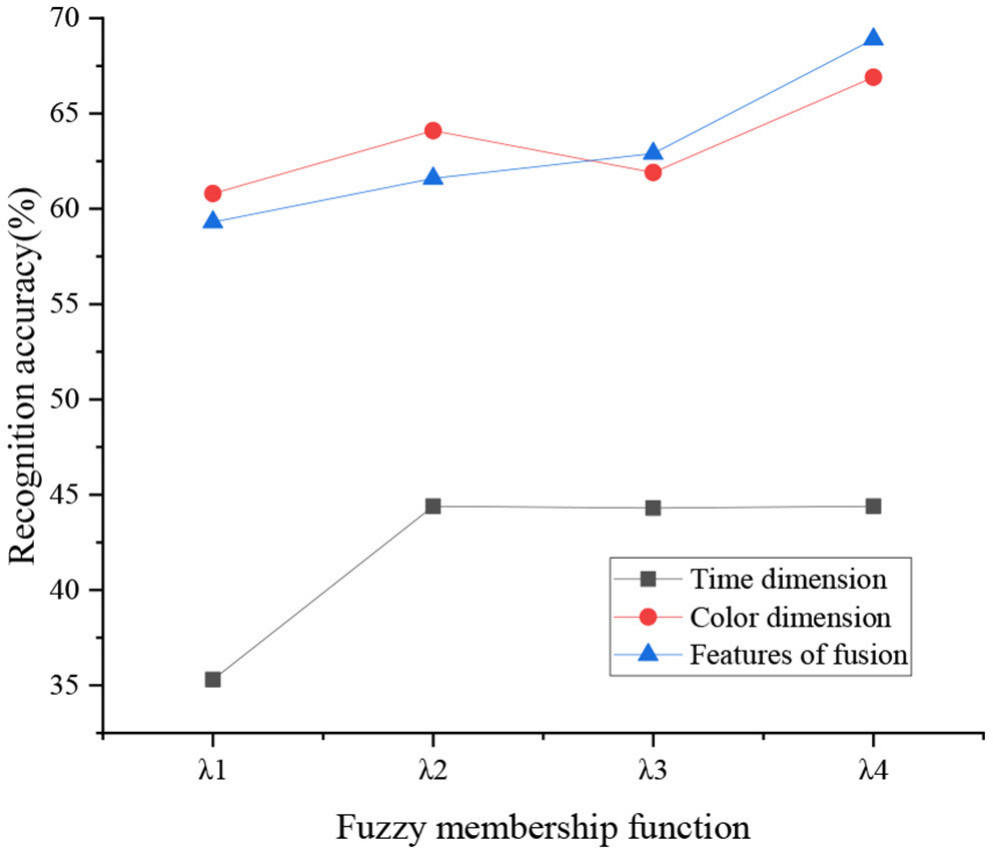
Comparison of micromotion processing capabilities of different algorithms.

Figure 11 denotes the recognition of different dimension features by fuzzy MF.

Figure 11 illustrates that the feature recognition rate of RGB features is higher than that of the time feature, by up to 24%. The recognition rate of the time dimension is low because the microhand motions are easy to overlap in movement. The first fuzzy MF has a lower recognition rate. The second, third, and fourth MFs have a better recognition rate because they represent the significance of the beginning, middle, and end of the video sequence. Overall, the accuracy of the RGB color feature is better than that of the time feature because microhuman motions are more likely to overlap. In this case, the recognition of color feature is higher. In the human body recognition research on the phenomenon of human body overlap, the recognition rate of the proposal of Santos et al. (2022) is consistent with the present work.

**Figure 12:**
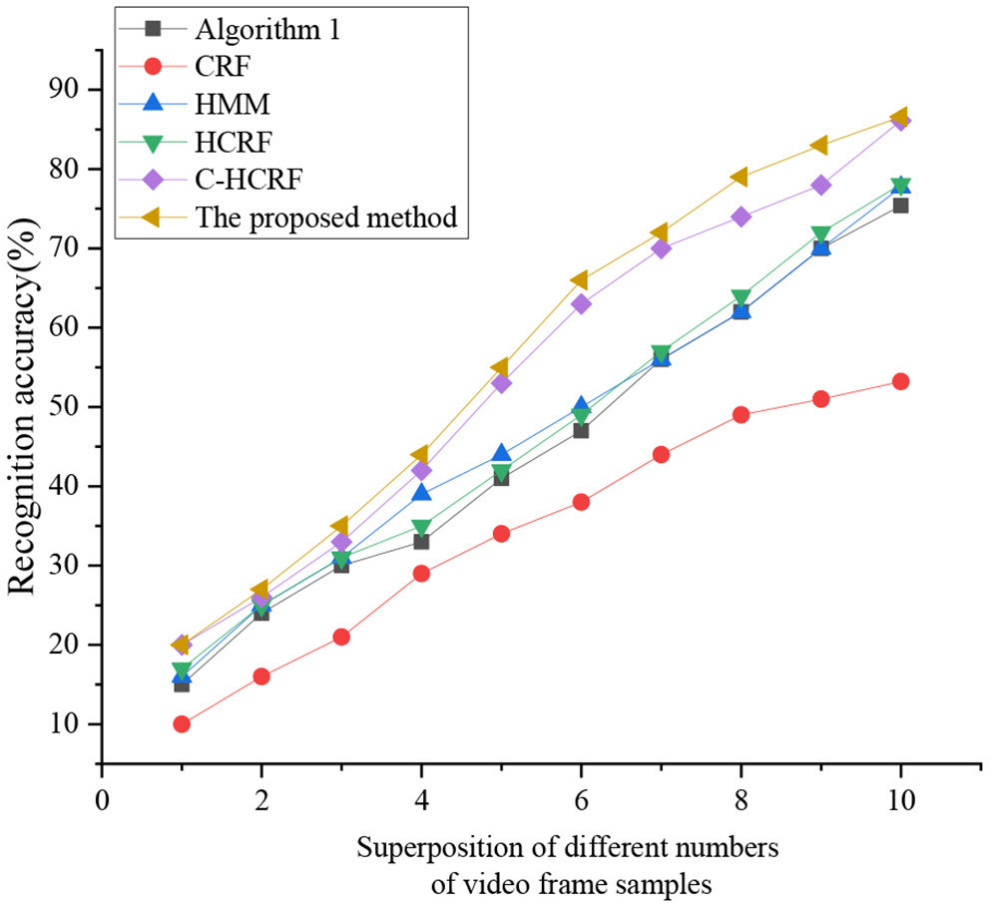
Comparison of accuracy of micromotion recognition with different algorithms.

Afterward, the last three fuzzy MFs are fused. The comparison is made between the proposed KELM-MFF-based HMR algorithm and the SVM-MFF-based HMR algorithm in recognizing micromotions, as sketched in Figure 12.

As Figure 12 displays, the proposed KELM-MFF-based HMR algorithm has the highest recognition rate of the other five algorithms. The proposed KELM-MFF-based HMR algorithm fuses motion information with RGB features. It has more advantages in capturing micromotion than the other five algorithms. Hidden Markov algorithm (HMM), algorithm 1, and conditional random field (CRF) belong to a single recognition algorithm, so the recognition rate is low. By comparison, the hidden conditional random field (HCRF) and the continuous hidden conditional random field (C-HCRF) use video sequences. They have a higher recognition rate because they belong to multidimensional recognition. Apparently, the proposed KELM-MFF-based HMR algorithm can be used to deal with micro-HMR in sports videos. Compared with the latest research results of Varshney et al. (2022), the experimental accuracy of the present work is higher, indicating the superiority of fusing motion information and color features. However, the analysis of color fusion in the literature is more in-depth than the present work, so the result findings are more convincing than the present work. Thus, the present work will also do more in-depth research on color fusion in the future.

### Comparison of Feature Fusion Strategies of Different Algorithms

**Figure 13:**
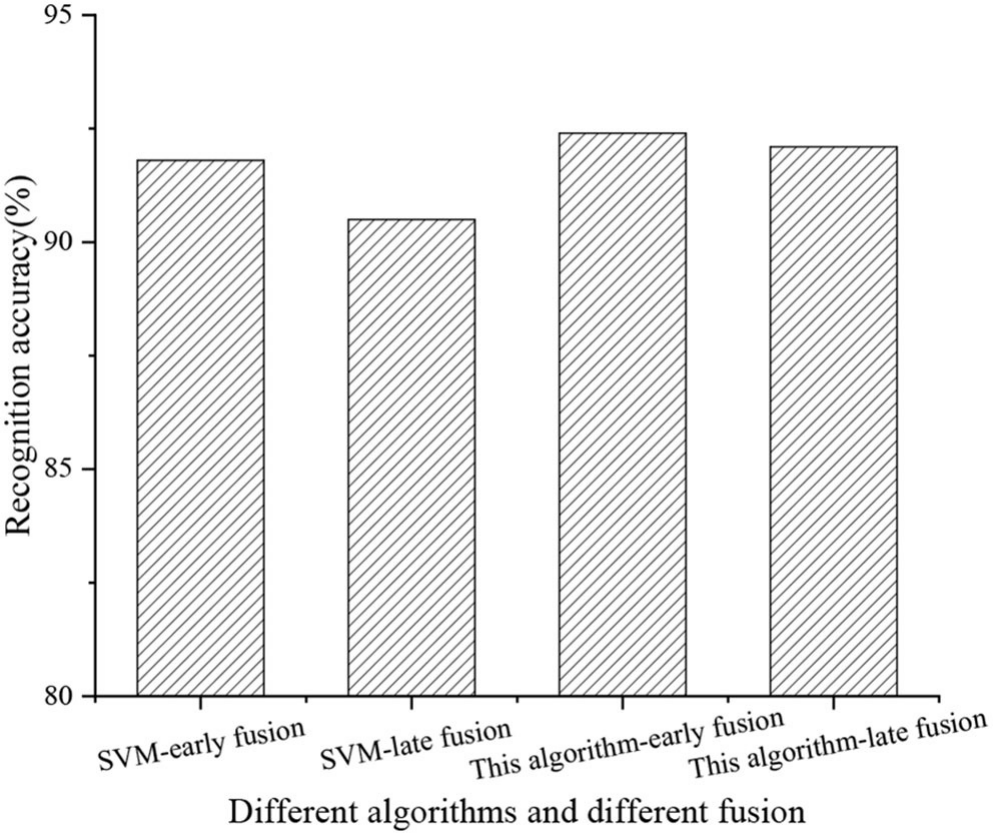
Comparison of recognition rate under different algorithm feature fusion strategies.

Figure 13 compares the recognition rate between different fusion strategies of the SVM-MFF-based HMR and the proposed KELM-MFF-based HMR.

Figure 13 signifies the recognition rate. Apparently, the proposed KELM-MFF-based HMR algorithm is higher than the SVM-MFF-based HMR algorithm in both the early and late feature fusion stages, reaching 92.4 and 92.1%. The SVM-MFF-based HMR algorithm has reached 91.8 and 90.5%. The conclusion draws that the recognition rate is higher when features are fused earlier than later under both algorithms.

### Comparison of Time Efficiency of Different Algorithms

**Figure 14:**
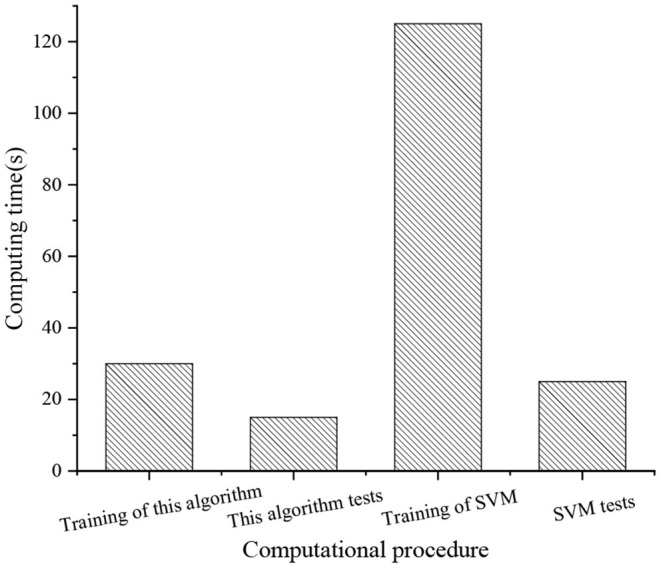
Comparison of time efficiency of different algorithms.

The time efficiency of the proposed KELM-MFF-based HMR algorithm is compared with that of the SVM-MFF-based HMR algorithm, as plotted in Figure 14.

Figure 14 signifies that the proposed KELM-MFF-based HMR algorithm takes a shorter time to train and test than the SVM-MFF-based HMR, only 30 and 15 s for training and testing. By comparison, the SVM-MFF-based HMR algorithm takes 125 and 25 s. Thus, the time efficiency of the proposed KELM-MFF-based HMR algorithm is much higher than that of the SVM-MFF-based HMR algorithm. Therefore, the proposed KELM-MFF-based HMR algorithm can be used to deal with large-scale datasets. Bhatia et al. (2022) also have observed that the KELM is suitable for processing large-scale datasets. At present, there are few researches on large-scale dataset processing in sports. The advantage of the present work is applying the proposed KELM-MFF-based HMR algorithm to the field of sports.

## Discussion

In improving HMR, the recognition rate of the proposed algorithm on large-scale data is more than 86%, higher than that of the SVM, CNN-T, IDT, C3D algorithm, and CNN algorithms. The recognition rate of the CNN-T algorithm is also high, which intuitively shows the superiority of the MFF algorithm. With the rapid development of science and technology, the combinatorial algorithm has become the first choice of current researchers. The MFF algorithm can improve the performance of a single algorithm and make up for the limitations of a single algorithm. The latest research by Kyaw et al. (2022) shows that the MFF algorithm is a critical way to solve practical problems. HMR is inseparable from color recognition. The accuracy of color recognition of the proposed algorithm is affected by the phenomenon of human action overlap. It is hoped to strengthen the research on improving the recognition rate of action overlap in the future. For HMR, the proposed KELM-MFF-based HMR has the highest accuracy among the five comparison algorithms, mainly because this algorithm integrates RGB color features and motion information. The research of Yang and Zou (2022) suggests that the integration of RGB color features plays a vital role in recognition algorithms. The present work verifies that the ELM is suitable for processing large-scale video datasets through the time efficiency comparison of different algorithms. The finding provides data support for applying the ELM algorithm in video recognition fields.

## Conclusion

Following a review of the HMR system using the DL algorithm, the present work studies the application of HMR systems in sports competitions. After background introduction, a KELM-MFF-based HMR algorithm is designed to improve traditional algorithms' poor performance against large-scale data and micromotions in sports videos. Then, a simulation experiment is designed to evaluate the performance of the proposed KELM-MFF-based HMR algorithm. The research findings corroborated that the proposed KELM-MFF-based HMR algorithm can be used to solve two problems in the current algorithm. (1) The DL features of human motions in the video sequence are analyzed through the time template to assign different significance to different time domains of the motion information. (2) The time template of the video sequence is inputted into the CNN to learn the feature set of sports motions. The manual and DL features are complementary and describe the human motions in videos from different angles. The research content provides a reference for applying the DL algorithm in sports competitions. There are still some deficiencies in the article. The second experimental dataset (NATOPS video dataset) only involves the professional motions in basketball and volleyball without adding other sports. Meanwhile, the analysis of color characteristics is not deep enough. The present work does not optimize the hyperparameters of the model. The follow-up research can combine the common sports actions into a new dataset for a more comprehensive analysis. There is a need to deepen the research on color characteristics and increase the hyperparameter setting and recognition rate of human actions for more convincing research results. After further improvement, it is expected to be applied to college sports events.

## Data Availability Statement

The raw data supporting the conclusions of this article will be made available by the authors, without undue reservation.

## Ethics Statement

The studies involving human participants were reviewed and approved by Xi'an Physical Education University Ethics Committee. The patients/participants provided their written informed consent to participate in this study. Written informed consent was obtained from the individual(s) for the publication of any potentially identifiable images or data included in this article.

## Author Contributions

The author confirms being the sole contributor of this work and has approved it for publication.

## Conflict of Interest

The author declares that the research was conducted in the absence of any commercial or financial relationships that could be construed as a potential conflict of interest.

## Publisher's Note

All claims expressed in this article are solely those of the authors and do not necessarily represent those of their affiliated organizations, or those of the publisher, the editors and the reviewers. Any product that may be evaluated in this article, or claim that may be made by its manufacturer, is not guaranteed or endorsed by the publisher.
